# Single‑pass four‑throw versus traditional knotting pupilloplasty for traumatic mydriasis combined with lens dislocation

**DOI:** 10.1186/s12886-023-02773-z

**Published:** 2023-01-09

**Authors:** Chaolan Shen, Lingjuan Liu, Ning Su, Ling Cui, Xin Zhao, Min Li, Haibin Zhong

**Affiliations:** Institute of Ophthalmic Diseases, Guangxi Academy of Medical Sciences & Department of Ophthalmology, the People’s Hospital of Guangxi Zhuang Autonomous Region & Guangxi Key Laboratory of Eye Health & Guangxi Health Commission Key Laboratory of Ophthalmology and Related Systemic Diseases Artificial Intelligence Screening Technology, Nanning, Guangxi People’s Republic of China

**Keywords:** Pupilloplasty, Ocular trauma, Mydriasis, Modified siepser technique

## Abstract

**Purpose:**

To compare the use of single‑pass four‑throw (SFT) and traditional double-pass two-throw knotting (DTT) techniques in pupilloplasty for traumatic mydriasis combined with lens dislocation, and to evaluate the learning curve between the two knotting techniques by wet lab.

**Method:**

The eyes of 45 patients (45 eyes) were divided into two groups according to the knotting technique used: single‑pass four‑throw (22 eyes) or traditional double-pass-two-throw knotting (23 eyes). Combined phacoemulsification and pupilloplasty with pars plana vitrectomy were performed in traumatic mydriasis patients with lens dislocation. Preoperative and postoperative corrected distance visual acuity (CDVA), pupil diameter, intraocular pressure (IOP), pupilloplasty time, and complications were compared. Twenty ophthalmology residents were randomized to perform a pupilloplasty suturing exam with or without SFT knotting techniques in porcine eyes.

**Result:**

All cases had a minimum follow‑up period of 6 months (range 6–12 months). There was no significant difference in the CDVA (*P* = 0.55), postoperative pupil diameter (*P* = 0.79), IOP (*P* > 0.05), anterior chamber exudate degree, and loosening or shedding of the line knot between the two groups. The duration of the pupilloplasty was 22.32 ± 4.58 min in the SFT group and 30.35 ± 5.55 min in the traditional group, which was a significant difference *(P* < 0.01). The residents in the SFT group had higher test scores and fewer surgical mistakes (*P* < 0.05).

**Conclusion:**

The SFT knotting technique has a similar treatment effect and safety as the traditional technique but requires a shorter time and is easier to perform in pupilloplasty surgery.

## Introduction

Traumatic iridodialysis caused by blunt eyeball trauma is often accompanied by lens dislocation. Blunt ocular trauma causes the sphincter pupillae muscle to stretch, posterior movement of the lens/iris diaphragm, and an acute elevation in intraocular pressure, and consequent tearing of the tissues near the anterior chamber angle. Abnormalities in pupil size, distortion, and eccentricity affect vision and cause symptoms such as photophobia, monocular diplopia, debilitating glare, and halos.

Medications for traumatic iridodialysis are ineffective; however, the condition can be repaired by pupilloplasty surgery. Currently, pupilloplasty technique is suitable for a wide range of applications, including iris abnormalities secondary to intraocular trauma or surgery, and angle‑closure glaucoma [1]. There are numerous different surgical techniques that employ sutures with different kinds of knots for pupil reconstruction and iris repair during pupilloplasty surgery. McCannel's method used for pupil reconstruction has been previously described, but it requires an extra limbal incision, and the modified McCannel's method or Siepser's method also require stitches to travel multiple times within the anterior chamber [2–4]. Priya Narang described that, in pupilloplasty, the single‑pass four‑throw technique [5] only requires one single pass compared to the modified Siepser technique.

Significant ocular blunt trauma necessitating iris reconstruction may also result in cataracts and sub- or complete dislocation of the lens. The usual treatment for a combined iris and lens capsule deficiency is iris prosthetic implantation via scleral sutures or iridoplasty with scleral-fixated IOL [6]. Intraocular lens (IOL) implantation in an aphakic eye without capsular support can be challenging. There are many reports of intraocular lens (IOL) fixation in eyes without sufficient capsular support. Representative techniques include anterior chamber IOL implantation, iris-fixed IOL implantation and trans-scleral fixed posterior chamber IOL implantation with or without suture [7–9]. The other approach is to apply contact lenses in a patient with aphakia [10].

The purpose of this retrospective study was to compare the clinical outcomes of 45 eyes with traumatic iridodialysis and traumatic lens dislocation treated by two knotting methods performed in pupilloplasty. In a wet lab, we further evaluated the learning curve of ophthalmology residents’ abilities to perform two knotting methods during pupilloplasty.

## Materials and methods

### Study design

We retrospectively assessed 45 traumatic mydriasis patients with lens dislocation who underwent pupilloplasty with phacoemulsification between August 2017 and June 2020 at the People’s Hospital of Guangxi Zhuang Autonomous Region. Patients who had major systemic or ocular surface diseases and a history of earlier ocular surgery or glaucoma were excluded.

Basic patient data, operative information, visual outcome, and complications were recorded. All patients underwent complete preoperative ophthalmic examinations, including corrected distance visual acuity, fundoscopy, intraocular pressure assessment, and slit-lamp biomicroscopy.

### Surgical technique

To ensure consistency, the same surgeon (Zhong Haibin) performed all the surgeries under an operating microscope. The surgical technique was described as follows. After administration of conventional retrobulbar anesthesia, all patients underwent 23G or 25G three-port pars plana vitrectomy. The dislocated lens was captured in the anterior chamber with the help of a chandelier fiber for extraction, and intraocular lens (IOL) implantation was performed (Alcon, CZ70BD) with upper and lower loop fixation. Pupilloplasty was performed if the pupil was still larger than 4 mm after 1.0 ml camcoline was injected into the anterior chamber (AC).

**Fig. 1:**
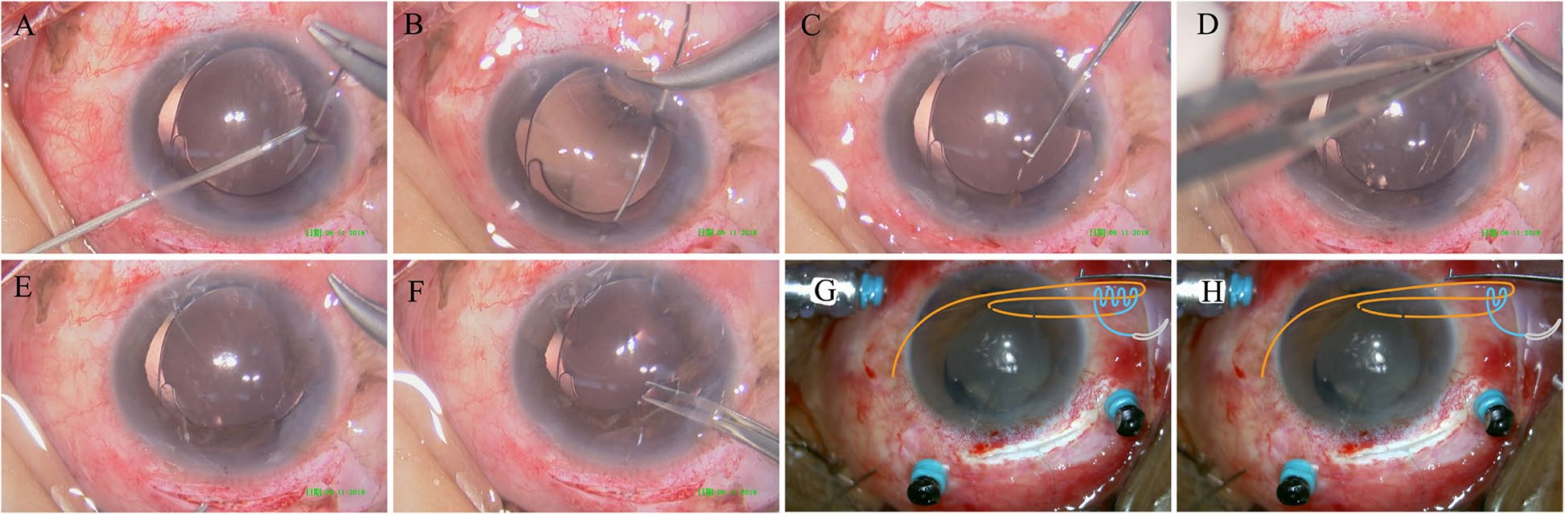
Illustration of the surgical steps of pupilloplasty surgery. A needle was inserted from the corneal limbus into the anterior chamber. The needle going through the iris was assisted using a microhook (**A**), and the needle went out from the opposite corneal limbus (**B**). A microhook was used to guide a loop of left sutures from the anterior chamber out through the main/assistant cataract incision (**C**). SFT or DTT methods were chosen to make a knot (**D**). smooth the loop, and then snip the end of the suture inside the eye (**E**/**F**). SFT, the suture end is passed through the loop 4 times (**G**). DTT, the suture end is passed through the loop 2 times (**H**)

Iris suturing was performed with 10–0 polypropylene sutures on a round-bodied needle (Alcon Laboratories, Inc., Sinking Spring, PA, USA). Na-HA (1.7%, Bausch & Lomb Co., Ltd., Shang Dong, China) 0.15- A total of 0.2 mL was injected into the AC to form sufficient operating space and protect the corneal endothelium. From the surgeon's right-hand corneal limbus, one incision was created along the axis of the area of iris defect, a needle was inserted into the AC through the incision (Fig. 1A), the iris of the pupil border was sutured to one quadrant around the entire pupillary margin into position, and the needle was removed from the left corneal limbus (Fig. 1B). Next, a microhook was used to guide a loop of suture from the opposite side of the anterior chamber out through the proximal incision (Fig. 1 C). SFT or DTT methods were chosen to make a knot (Fig. 1 D). Both ends of the suture were pulled to internalize the knot (Fig. 1 E). Thus, both edges of the iris were tightened. Both ends of the suture were snipped inside the eye (Fig. 1F). Knotting details of STF and DDT are described as follow: (1) The distal end of the suture is then pulled in the same direction through the loop until 4 throws are taken, with each throw passing through the loop (STF, Fig. 1 G). (2)The suture tail is passed in the same direction through the loop until 2 throws are taken. The same technique was repeated to fix the knot (DTT, Fig. 1 H). Usually, 1–2 stitches were placed on the pupil superonasal and supertemporal, subnasal and subtemporal regions. After the operation, the pupil is round or almost round, the size is approximately 2.5 ~ 4.0 mm, and then the final suture of the corneoscleral incision is performed.

### Postoperative follow-up and complications

Patients were followed up on postoperative Days 1, 3, and 14 and then at months 1, 3, 6, and 12. All cases completed at least 6 months of follow-up postoperatively (range 6–12 months). At each visit, CDVA and IOP were measured, and slit-lamp biomicroscopy and fundoscopy examinations were performed.

To assess the anterior chamber inflammation of the 2 groups, the anterior chamber (AC) flare was graded according to the grading system defined by the Standardization of Uveitis Nomenclature (SUN) Working Group criteria [11]. Thus, grade 1 indicated that the AC flare was faint, grade 2 indicated moderate (iris and lens details clear), and grade 3 indicated marked (iris and lens details hazy). Grade 4 indicated intense (fibrin or plastic aqueous).

The postoperative symptom evaluations were assessed by an ophthalmologist (Chaolan Shen) at each postoperative examination until the second week.

### Learning curve assessment by the wet lab

Twenty residents and 2 anterior segment attendings were recruited from the People’s Hospital of Guangxi Zhuang Autonomous Region Department of Ophthalmology. We studied the learning curve of the STF and DDT knotting modalities for resident microsurgical education through a wet lab. Residents were randomized into 2 groups, STF and DTT. The residents participated in a lecture that reviewed the proper suturing technique and explained the pupil suturing task, which consisted of passing 1 cardinal suture of the pupil border in porcine eyes with iridodialysis using 10–0 polypropylene sutures. The residents were provided 1–3 practice courses, which were performed at their discretion, before the suturing task. Each suture pass was objectively scored by 2 anterior segment attendings using the criteria shown in Table 1 to record operational mistakes.

**Table 1:** Suture test score deduction criteria

Criteria	Points (20 basic score)
Extra enter anti chamber	-1
Loosening of the loop	-2
Corneal tunnel incision leak	-1

### Statistical analysis

Statistical analyses were carried out with SPSS software (version 21.0; SPSS, Chicago, IL). T tests, chi-square tests and Fisher’s exact probability tests were used for the statistical analyses. A *P* value less than 0.05 was considered statistically significant.

## Results

Overall, the eyes of 45 patients (45 eyes) were divided into two groups according to the knotting technique used: STF (22 eyes) or DTT (23 eyes) in pupilloplasty combined with phacoemulsification for traumatic mydriasis. All cases had a minimum follow‑up period of 6 months (range 6–12 months) (Table 2).

**Table 2:** Demographic and clinical details

Group(n)	Age(year)	Male	DCVA LogMAR		Pupil diameter		Exudate grade	Pupilloplasty time
			Pre	Post	Pre	Post		
SFT (22)	43.32 ± 13.79	15	1.27 ± 0.293	.620 ± 0.404	6.62 ± .978	3.02 ± .516	I(8)II(10)III(5)	22.32 ± 4.58
DTT (23)	39.57 ± 11.35	17	1.29 ± 0.314	.687 ± 0.358	6.84 ± .90	2.99 ± .420	I(6)II(12)III(4)	30.35 ± 5.55
	*P* = 0.33	*P* = 0.46	*P* = 0.55		*P* = 0.79		*P* = 0.82	*P* < 0.01

The surgical duration of the pupilloplasty was 22.32 min in the SFT group and 30.35 min in the DTT group, which was a significant difference (*P* < 0.01).

There was a significant increase in CDVA after the operation in both groups. Logmar CDVA significantly improved from 1.29 to 0.68 in the SFT group (*P* < 0.01) and improved from 1.27 to 0.62 in the DTT group (*P* < 0.01). There was no significant difference between the groups in the CDVA (*P* = 0.55).

**Fig. 2:**
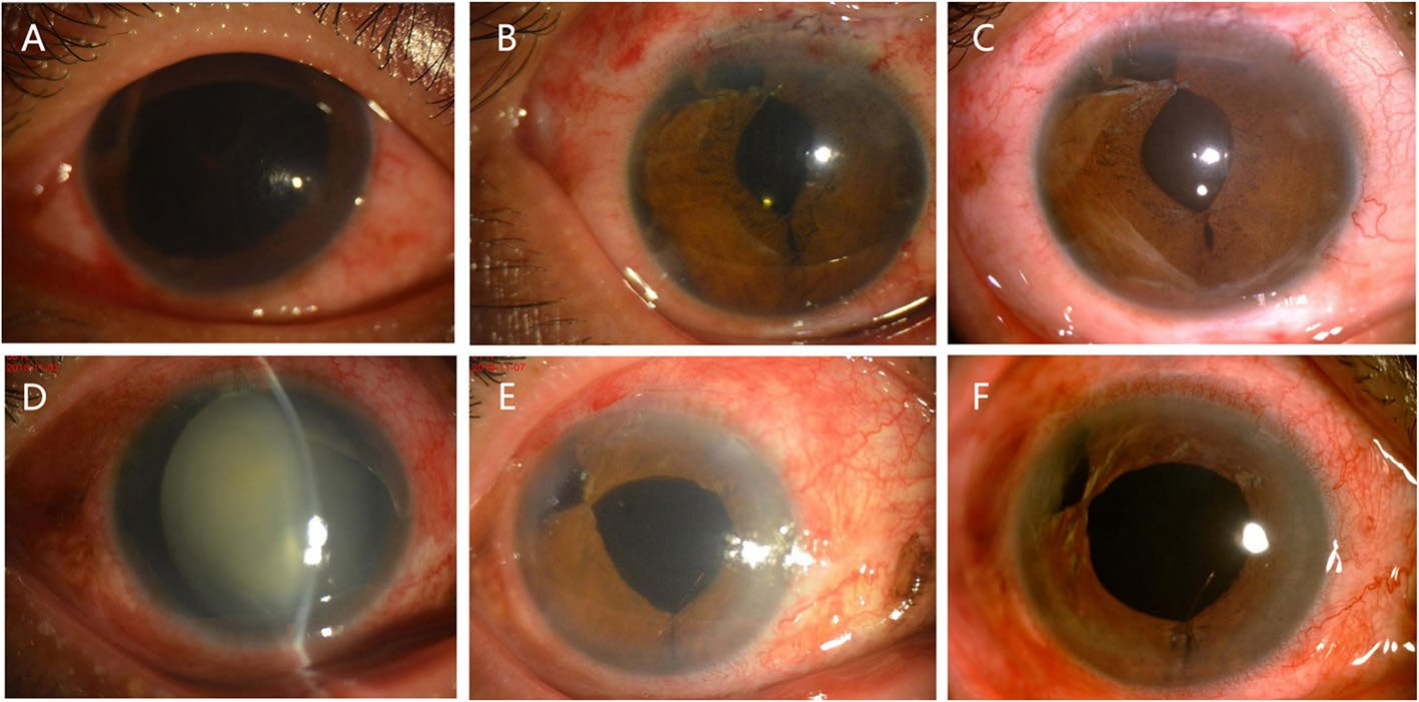
Pictures taken on preoperative (**A**) and postoperative Days 3 (**B**) and 3 months (**C**) of a patient in the SFT group. Preoperative (**D**) and postoperative Day 3 (**E**) and 3 months (**F**) of a patient in the DTT group

There was a significant reduction in postoperative pupil diameter after the operation in both groups. The postoperative pupil diameter decreased from 6.62 to 3.02 in the SFT group (*P* < 0.01) and from 6.84 to 2.99 in the DTT group (*P* < 0.01). There was no significant difference in the postoperative pupil diameter between the two groups (*P* = 0.79). The pre- and postoperative appearances of the patients in both groups on Day 3 and at 3 months are shown in Fig. 2.

There was no significant difference in the anterior chamber exudate degree between the groups (*P* = 0.82). An exudate of the anterior chamber was common in the early postoperative period, and exudate absorption was observed after anti-inflammatory treatment. There was no significant difference in intraocular pressure before or after the operation between the groups (*P* > 0.05). Four patients in both groups had iris tears during the suture operation, and 3 had temporary incidences of high IOP postoperation, which were controlled by antiglaucoma drugs. In the DTT group, suture slippage was observed at the 1-month postoperative follow-up.

Twenty residents were recruited to participate in a wet lab training course and randomized into two groups according to the knotting technique. Table 3 summarizes the baseline resident characteristics for the SFT and DTT groups.

**Table 3:** Baseline resident characteristics and test performance

Group(n)	DTT (10)	STF(10)
Age ± SEM, y	27.6 ± 2.0	27.8 ± 1.5
Sex, M/F	3/7	4/6
postgraduate year		
PYG -1	4	3
PYG-2	3	3
PYG-3	3	4
training time	1(0)2(3)3(7)	1(1)2(5)3(4)
suture time(min)	40.5 ± 4.9	31.5 ± 7.0
score	17.7 ± 1.4	19.1 ± 1.3
Loosen loop	3	1
Extra enter AC	13	3
Corneal tunnel incision leak	4	4

*PGY* postgraduate year, *SEM* standard error of the mean

**Fig. 3:**
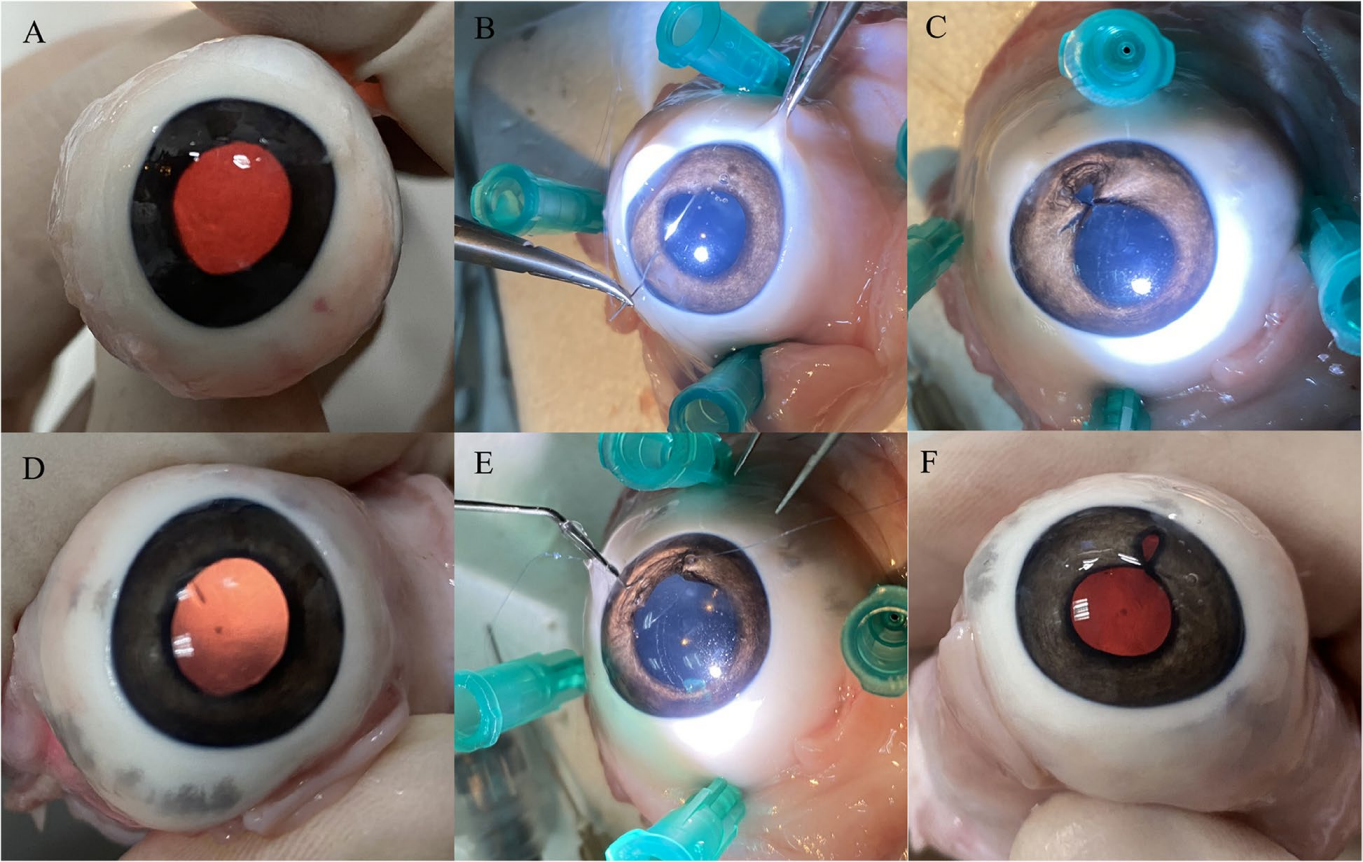
Pictures of the suture test using porcine eyes. Preoperative (**A**), Intraoperative (**B**) and Postoperative (**C**) pupilloplasty was performed on porcine eyes in the SFT group. The operation process in the DTT group (**D**) (**E**) (**F**)

After taking hearing a lecture and taking a practice course, the residents participated in a suture test of the pupil border in a porcine with iridodialysis using 10–0 polypropylene sutures (Fig. 3). At the conclusion of the wet lab suture exam, 2 anterior segment attendings were identified using the suture test score deduction criteria.

**Fig. 4:**
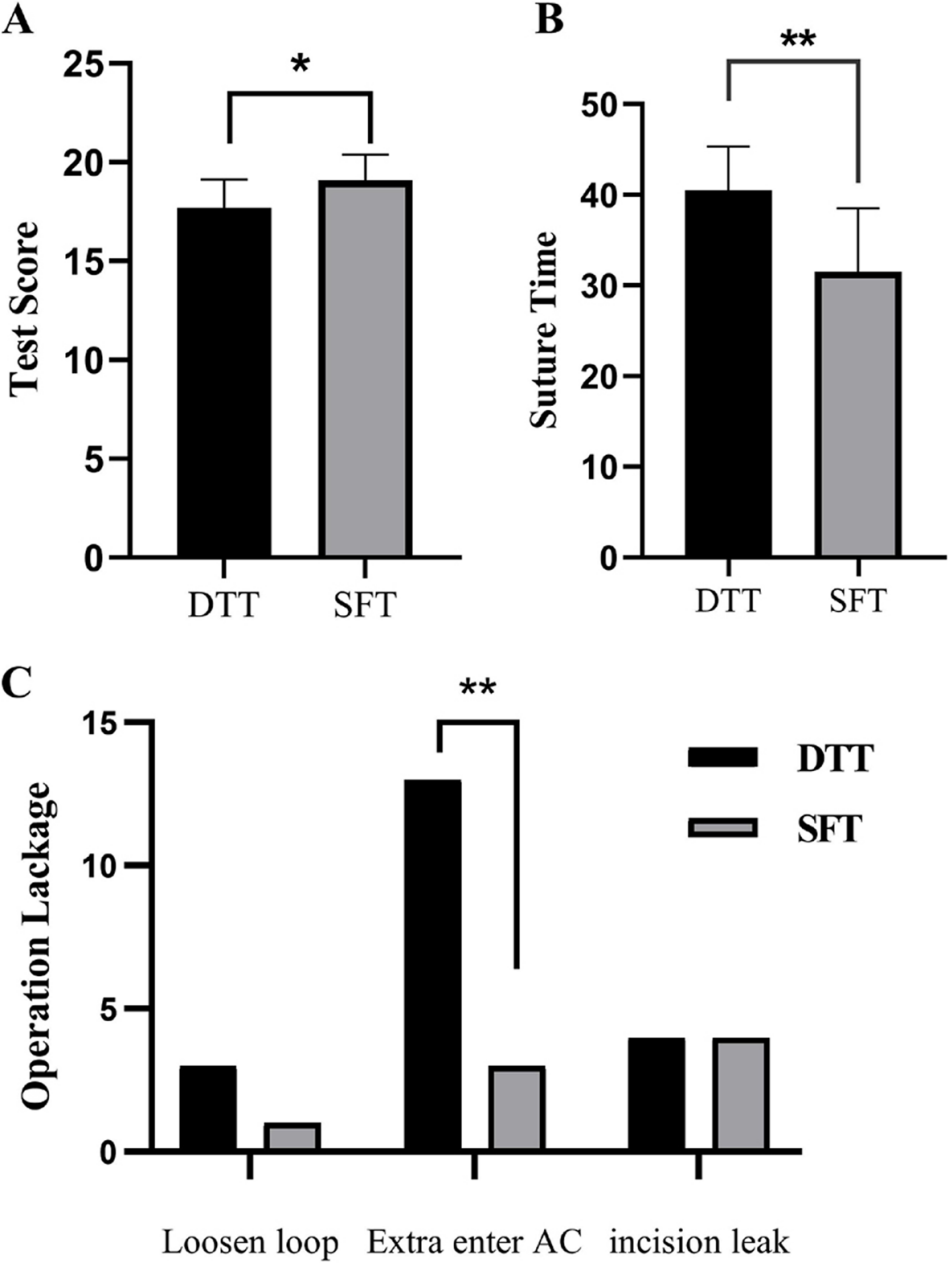
**A** Mean suturing scores for residents in the SFT and DTT groups. The SFT group (19.1 ± 1.3) performed better than the DTT group (17.1 ± 1.4). **B** The suturing performance of the SFT group was significantly shorter than that of the DTT group. These surgical faults were only statistically significant for additional entrances into the AC. *P < 0.05; **P < 0.01

Figure 4 highlights objective pupilloplasty suturing performance results for the SFT and DTT groups. The SFT group required significantly less suture time than the DTT group (*P* < 0.01). Residents in the SFT group avoided entering the anterior chamber significantly more often than those in the DTT group (*P* < 0.01). The SFT group had better overall test scores and performed significantly better than the DTT group (*P* < 0.05).

## Discussion

The pupil is an essential component of the optic system of the human eye. Pupil deformation is associated with several manifestations of vision degradation, including photophobia, monocular diplopia, peripheral aberrations, and light scattering, all of which can reduce optical quality. In 1976, McCannel tried to perform an iris suture during extracapsular cataract surgery, which resulted in improved function of the iris from weakened to recoverable [4]. Therefore, the aim of pupilloplasty surgery is to restore the pupil to an appropriate size, which is critical for high visual performance [12]. However, some complex penetrating ocular trauma cases can be associated with other pathologies, such as corneal scars, glaucoma, traumatic cataracts, and posterior segment complications. Iris prosthetic implantation [13], contact lenses, and ocular prostheses can be used in eyes with iris tissue defects that are ineligible for pupilloplasties to improve visual acuity and achieve good esthetic results. Often times, these eyes must undergo more than one surgery until final rehabilitation is achieved. A careful, post-trauma surgical strategy should be established for globe reconstruction using secondary lens implantation.

The iris suture surgical method has been continuously improved [14, 15]. In the past ten years, our team has performed the modified Siepser slip knot technique for pupil reconstruction, which has been previously described [3]. An approximating loop is taken with either 2 or 3 throws followed by a securing loop that helps to tie the knot. However, in recent years, Narang and Agarwal has described a new iris suture knot [5]. A single-pass 4-throw (SFT) technique is performed by intertwining the thread around itself, which acts as a lock mechanism and ensures nonloosening of the loop. Our study confirmed the safety and efficiency of the SFT technique compared to the traditional double-pass two-throw knotting (DTT) technique.

According to our results, the duration of pupilloplasty surgery in the SFT group was significantly shorter. A longer duration of surgery makes the patient feel tired and uncomfortable. Another advantage of SFT is that it reduces the number of times the microhook enters the AC; on average, the hook enters one time for each knot. Each time the anterior chamber is breached, the risk of infection and corneal endothelial cell loss increases. Endophthalmitis rates were found to be substantially higher among patients undergoing cataract surgery combined with other ocular procedures [16]. The result was also confirmed by a wet lab comparison.

In our study, there were no significant differences in knot slippage after surgery between the two groups, and only one eye in the DTT group had knot slippage during the 1-month follow-up. We speculated that the same direction of double knots may form a sliding knot. The surgeon should be able to identify the parallel configuration of the polypropylene sutures, thus confirming the correct orientation of the sutures inside the anterior chamber, which may lead to an increased risk of a sliding knot among unskilled surgeons. Moreover, simplifying the knotting technique can significantly circumvent the challenges of learning this surgical skill. The SFT group had better overall performances in pupilloplasty suturing. There was a performance difference in surgical time and AC entrance times between the two groups. Taking care to avoid breaching the AC can significantly reduce surgical time and complications.

There were no significant differences in the iris tear rates during suturing between the two groups because the modified Siepser knotting technique was performed in situ, which reduced the excessive pulling on the iris. Additionally, the chance of tearing is low if the iris tissue has sufficient elasticity and if the instrument provides auxiliary traction to flatten the iris tissue, which is convenient for determining the appropriate suture point. However, we should not require pupils to be round and accurate in size, and it is not necessary to completely suture outside the eyelid fissure area due to the upper and lower eyelid margin cover effect. The postoperative symptom evaluations showed that the AC exudate grade between the groups was not significantly different, and all symptoms could be controlled by cortisol eye drops within one or two weeks. According to our results, we found similar postoperative CDVA and pupil diameter recovery outcomes between the SFT and DTT groups at the 6-month follow-up. Compared with DTTs, the patients who received SFTs seemed to have no serious complications during the follow-up period. Decreasing the time to AC can prevent extra corneal damage. In porcine surgical practice, comparison data showed that the SFT group had a significant reduction in the time to enter the AC, which prevented intraoperative complications.

## Conclusions

In conclusion, using SFT in pupilloplasty with phacoemulsification surgery for traumatic mydriasis patients with lens dislocation is safe, fast, and simple. SFT is a simple knotting process that reduces the surgery time, and is an easy suturing skill to master.

There were some limitations to this study. Although 22 to 23 patients were enrolled in each group, there was insufficient statistical power to accurately detect recurrences, as the risk was 5%. Additional studies with a larger sample size are needed to confirm the results of this study.

## Data Availability

The datasets used and/or analyzed during the current study are available from the corresponding author on reasonable request.

## Abbreviations

SFT: Single‑pass four‑throw
DTT: Traditional double-pass two-throw knotting
DCVA: Best corrected visual acuity
IOP: Intraocular pressure
IOL: Intraocular lens
AC: Anterior chamber
PGY: Postgraduate year
SEM: Standard error of the mean
